# Phenotypic Characterization Analysis of Human Hepatocarcinoma by Urine Metabolomics Approach

**DOI:** 10.1038/srep19763

**Published:** 2016-01-25

**Authors:** Qun Liang, Han Liu, Cong Wang, Binbing Li

**Affiliations:** 1 First Affiliated Hospital, Heilongjiang University of Chinese Medicine, Heping Road 24, Xiangfang District, Harbin 150040, China; 2 Simon Fraser University (SFU), Burnaby, British Columbia, Canada

## Abstract

Hepatocarcinoma (HCC) is one of the deadliest cancers in the world and represents a significant disease burden. Better biomarkers are needed for early detection of HCC. Metabolomics was applied to urine samples obtained from HCC patients to discover noninvasive and reliable biomarkers for rapid diagnosis of HCC. Metabolic profiling was performed by LC-Q-TOF-MS in conjunction with multivariate data analysis, machine learning approaches, ingenuity pathway analysis and receiver-operating characteristic curves were used to select the metabolites which were used for the noninvasive diagnosis of HCC. Fifteen differential metabolites contributing to the complete separation of HCC patients from matched healthy controls were identified involving several key metabolic pathways. More importantly, five marker metabolites were effective for the diagnosis of human HCC, achieved a sensitivity of 96.5% and specificity of 83% respectively, could significantly increase the diagnostic performance of the metabolic biomarkers. Overall, these results illustrate the power of the metabolomics technology which has the potential as a non-invasive strategies and promising screening tool to evaluate the potential of the metabolites in the early diagnosis of HCC patients at high risk and provides new insight into pathophysiologic mechanisms.

Metabolomics, which can be defined as measurement of the levels of all cellular metabolites, is the comprehensive assessment of endogenous metabolites and attempts to systematically identify metabolites from biological samples^1^. It attempts to capture global changes and overall physiological status in biochemical pathways in order to elucidate sites of perturbations, and has shown great promise as a means facilitate biomarker discovery from diseased patients^2^,^3^. Metabolomics has been applied to disease diagnosis, drug discovery, nutrition and toxicological studies, hold promise for discovery of pathways linked to disease processes^4^,^5^. It has emerged as a powerful tool for discovering new low molecular weight biomarkers and its utility has been demonstrated by the identification of disease-related biomarkers for prostate cancer, Parkinson’s disease, type 2 diabetes mellitus, acute myocardial ischemia and preeclampsia^6^,^7^,^8^.

Hepatocarcinoma (HCC) is the fifth most common cancer worldwide and third leading cause of death from cancer^9^. Approximately 564,000 new cases are diagnosed annually, including 398,000 men and 166,000 women^10^. Currently, patients with suspected HCC are initially subjected to liver function tests that include assessment of the serum levels of aspartate transaminase, alanine transaminase and glutamyl transpeptidase^11^,^12^. The only established tumor marker of HCC is serum R-fetoprotein^12^. Unfortunately, R-fetoprotein has a low sensitivity and specificity, and international guidelines have therefore concluded that R-fetoprotein is an inadequate surveillance test for HCC^13^. These considerations lead to a strong recommendation of the need for an effective biomarker of HCC, so that treatment may be optimized. Urine has been shown to contain a wealth of metabolic information that may be altered due to diseases, and do satisfy the criteria of minimal invasiveness, reasonable cost, or minimal time demand^14^,^15^. Therefore, finding urine biomarkers would be doubly advantageous because of its relatively noninvasive procedure and ability to provide an accurate and desirable diagnosis.

The conventional chemistry and histopathology method are not region-specific and only increase significantly after substantial disease injury. Therefore, more early markers of disease are needed. Fortunately, the rapid development of metabolomics technology has been used to explore the particular metabolites, potentially diagnostic and prognostic biomarkers of diseases. It has been used to characterize metabolic signatures for various diseases including depression^16^, Parkinson’s disease^17^, cancers^18^, diabetes^19^, cancer^20^ and Alzheimer’s Disease^21^. In a study, Zeng J *et al.* had used serum metabolomics method to discover novel biomarkers for HCC^22^. Tryptophan, glutamine and 2-hydroxybutyric acid was found based on the comprehensive screening workflow, highlighting the potential of early diagnosis. Their study also demonstrated the potential of metabolomics method in finding biomarkers for disease diagnosis and metabolism. Although a small number of metabolomic studies on HCC have been reported^23^,^24^,^25^,^26^, but metabolomic signatures on urine fluid of HCC has not yet been explored. Consequently, this paper was designed to investigate urine metabolome of HCC by LC-Q-TOF-MS combined with pattern recognition methods, in order to establish specific metabolites phenotype and explore the diagnostic possibilities.

## Results

### Analysis of metabolic pattern

**Figure 1:**
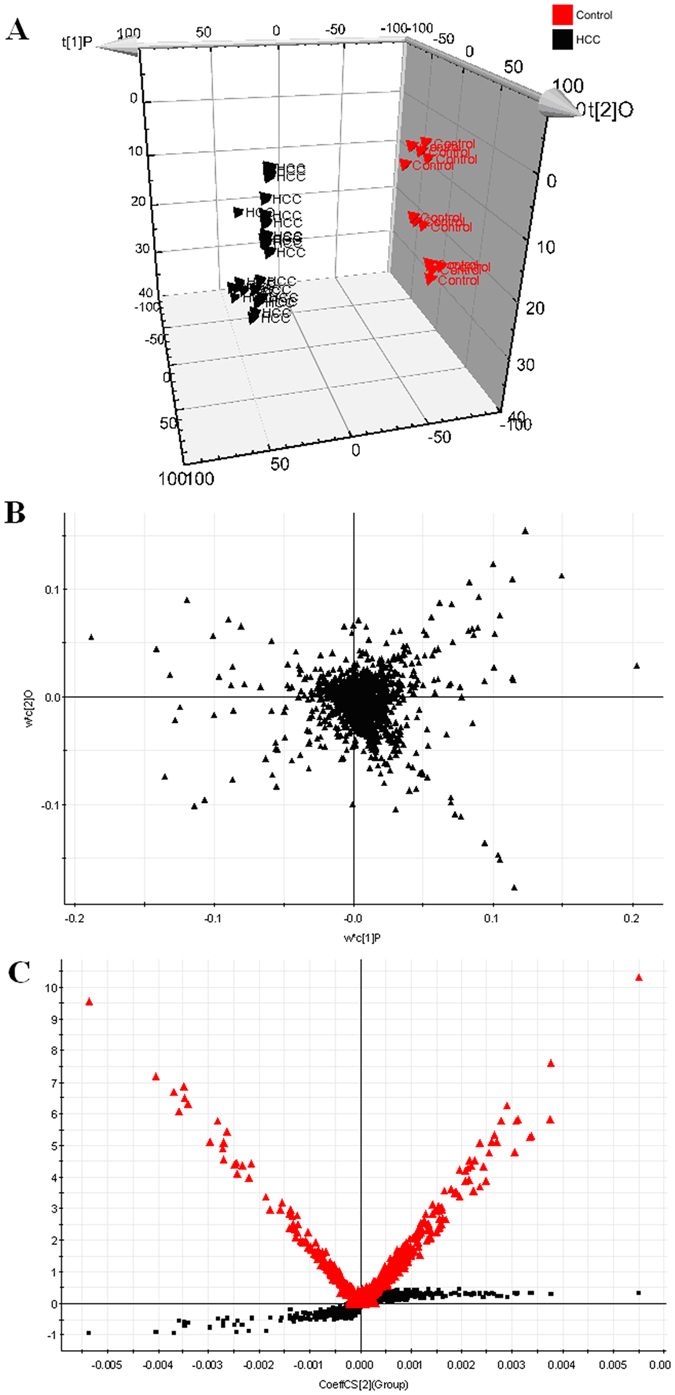
Metabolomic profiling of HCC in positive mode. 3-D of PCA model that is an unsupervised method of extracting information for HCC group (**A**). Loading plot of OPLS-DA of HCC in positive mode (**B**). Panel (**C**) shows the combination of S- and VIP-score plots constructed from the supervised OPLS analysis of urine (ESI+ mode). Ions with the highest abundance and correlation in the HCC group with respect to the controls are present on the upper far right hand quadrant, whereas ions with the lowest abundance and correlation in the HCC group with respect to the control group are residing in the lower far left hand quadrant.

**Figure 2:**
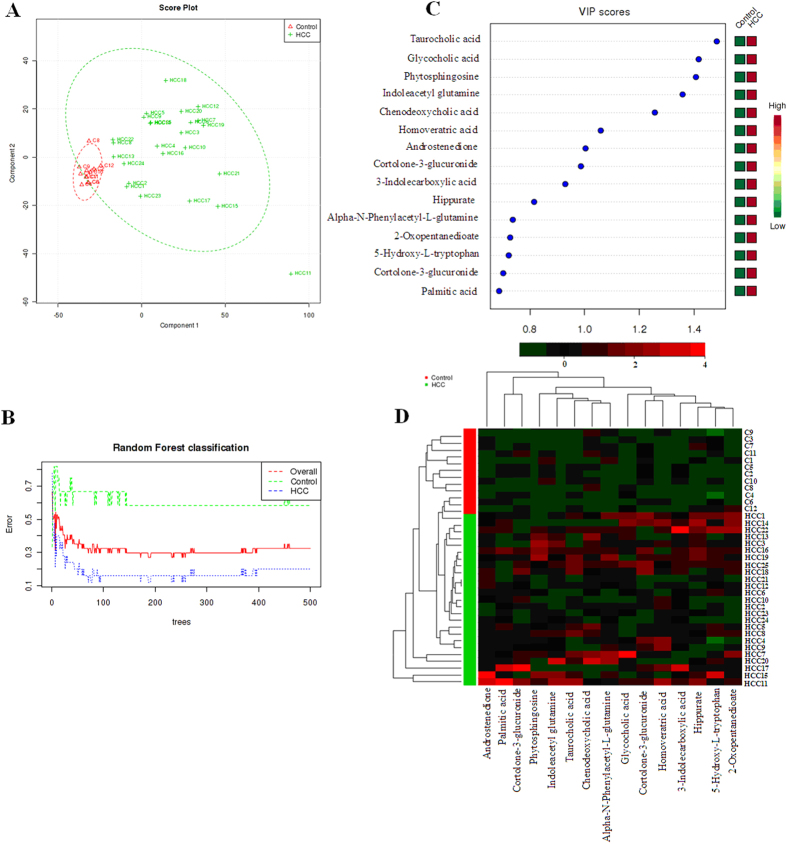
Systems analysis of metabolomic alterations of the control and HCC samples utilizing MetaboAnalyst’s data annotation tools revealed differences between the two groups. (**A**) Trajectory analysis of PCA Score plots for the HCC. (**B**) Random Forests machine learning algorithm classification for the HCC, implemented in MetaboAnalyst and used for unsupervised clustering. (**C**) Top 15 significant features of the metabolite markers based the VIP projection, which were extracted with random Forests analysis. (**D**) Heat map visualization and hierarchical clustering analysis for the urine of HCC. The heatmaps were constructed based on the top fifty metabolites of importance, which were extracted with random Forests analysis. Variable differences are revealed between the control and HCC groups, with verified and known ions marked on the bottom corresponding to SI Table 2. Rows: samples; Columns: metabolites; Color key indicates metabolite expression value, green: Lowest, red: highest.

**Figure 3:**
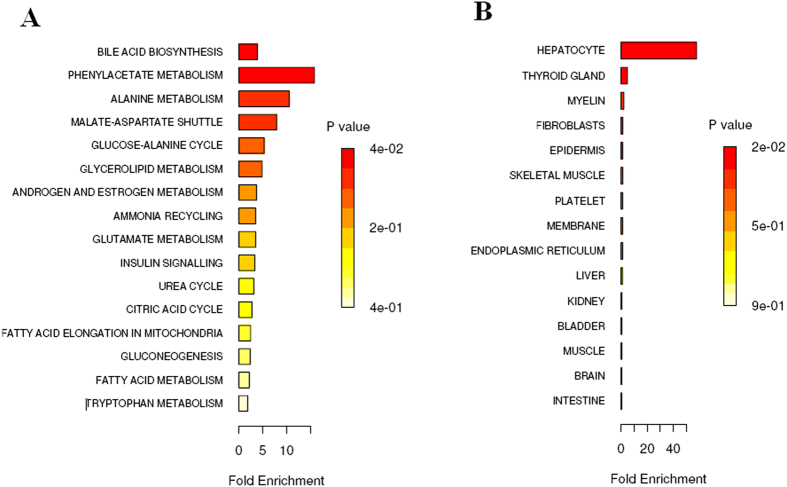
Summary Plot for Metabolite Set Enrichment Analysis (MESA) of HCC. (**A**) Pathway-associated metabolite sets for HCC. (**B**) Location-based metabolite sets for HCC.

To evaluate the capability of the LC-MS based metabolomic approach that is useful to differentiate HCC patients from controls, multivariate data analysis, machine learning algorithm approaches, were carried out in the study. Trajectory analysis of unsupervised PCA score plots (3-D) showed obvious separation between the HCC groups and healthy group in both positive (Fig. 1A) and negative ion modes (Supporting information (SI) Fig. 1A). From the corresponding the loading plots, the ions furthest away from the origin contribute significantly to be responsible for the separation between control and HCC groups and may be therefore regarded as the differentiating metabolites for HCC groups (Fig. 1B, and SI Fig. 1B). Combining the results of S- and VIP- plots from the OPLS analysis (Fig. 1C, and SI Fig. 1C), the LC-MS analysis platform provided the retention time, precise molecular mass and MS/MS data for the structural identification. A Wilcoxon Mann-Whitney test was performed and it was found that 15 ions (VIP > 6) significantly changed (p < 0.05) between the disease and the control groups. Finally, differential metabolites of significant contribution were characterizated and listed in SI Table 2. Trajectory analysis of PCA score plots and Random Forest classification for the HCC, implemented in MetaboAnalyst data annotation tools and used for unsupervised clustering, revealed differences between the two groups (Fig. 2A,B). Figure 3C showed top significant features of the metabolite markers based the VIP projection, which were extracted with Random Forests analysis. The potential candidates for biomarkers with OPLS was highly consistent with that parallel Random Forests and allow as depicted through two completely different algorithms for the ability to verify biomarkers. The parallels Heatmap (Fig. 2D), commonly used for unsupervised clustering, were constructed based on the top fifty metabolites of importance for the HCC and controls showed distinct segregation.

### Selection and Identification of important differential metabolites

To discover the potential biomarkers among thousands of variables, the following steps were then employed. The S-plot, a visualized method that can be applied to explain the variable influence on the model, could be helpful for the interpretation of metabolomic data, especially for filtering interesting metabolites in the projection, so that the risk of false positives in the metabolite selection procedure could be lowered. According to the variable importance in the projection (VIP) using the above OPLS-DA model, a total of 15 variables (ions) with a VIP > 6 were selected. It was observed that these metabolites could also be found in the dataset from the human metabolic profiling. The detailed method for the compound identification has been mentioned in our previous work^14^. The final results of the identified differential metabolites were shown in SI Table 2.

### Identification of metabolic pathways and enrichment analysis

In order to identify possible pathways that were affected by HCC, ions contributing to the separation of HCC were analyzed using Metabolite Set Enrichment Analysis (MESA), a way to identify biologically meaningful patterns that are significantly enriched in metabolomic data. Of the 16 assigned pathways, 4 were significant at a permutation value of p < 0.05 (Fig. 3A, SI Table 3). The significant pathways were bile acid biosynthesis, citric acid cycle, tryptophan metabolism, and urea cycle pathway. Location-based metabolite sets for HCC from MESA was found mainly in liver, hepatocyte, fibroblasts, kidney (Fig. 3B, and SI Table 4). Four (taurocholic acid, chenodeoxycholic acid, glycocholic acid, hippuric acid) of the 15 most significant metabolites were found to be belonged to bile acid biosynthesis pathway. The detailed construction of the altered bile acid biosynthesis metabolism pathways with higher score in human was generated using the reference map by searching KEGG and MassTRIX. Specifically, the bile acid biosynthesis pathway containing the significant metabolite taurocholic acid, chenodeoxycholic acid, glycocholic acid, hippuric acid was identified as the most significantly altered pathway in terms of individual metabolites’ regulation between HCC and control patients, received a significant functional score p-value at a value of p < 0.05.

### Validation of the marker metabolites

**Figure 4:**
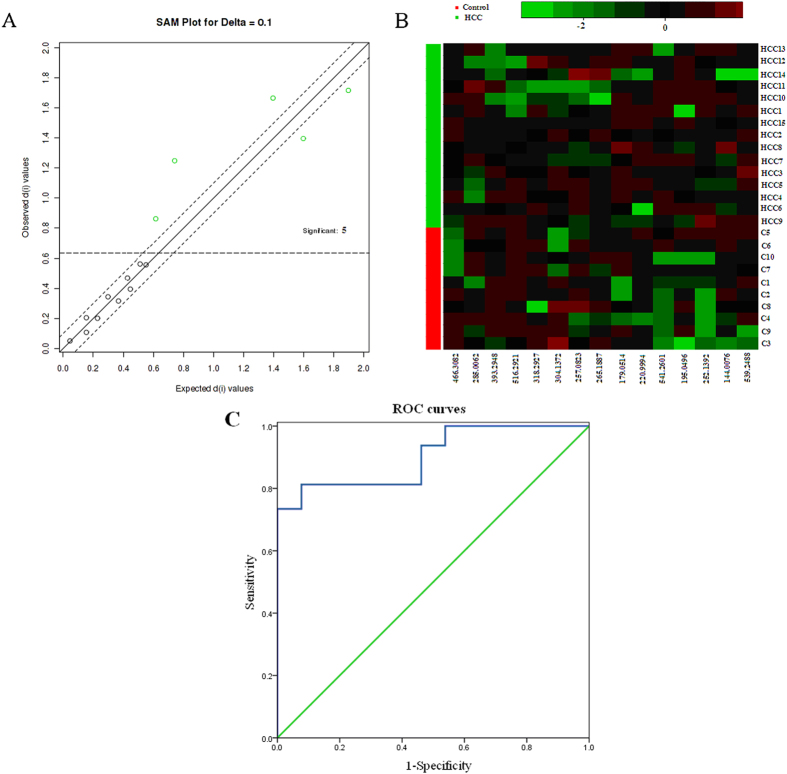
The selection and diagnostic potential of the most significant metabolites. (**A**) The significance analysis for microarrays method used to select marker metabolites. (**B**) The unsupervised clustering dendrogram of potential marker metabolites, tested our approach using a second set of HCC (n = 15) patients and control subjects (n = 10) to be blindly selected. (**C**) ROC curves for the diagnosis of HCC patients using metabolic markers.

To ascertain whether those significant urinary metabolites that ultimately may turn out to be biomarkers for HCC, we examined the metabolites that lie in nodes of KEGG pathway. The machine learning algorithms such as SAM do indicate that palmitic acid, alpha-N-Phenylacetyl-L-glutamine, phytosphingosine, indoleacetyl glutamine, and glycocholic acid were the most significant differential metabolites for the classification of the HCC and the control (Fig. 4A). To test the usefulness of the 5 marker metabolites for human HCC diagnosis, especially discriminating HCC from controls. A second set of HCC (n = 15) patients and control subjects (n = 10) to be blindly selected and tested using our metabolomics platform (Fig. 4B). To validate the significance of marker metabolites as a potential HCC biomarker, receiver operating characteristic curve (ROC) is plotted assuming the levels of marker metabolites in HCC group. Area under the curve (AUC) calculated using the trapezoidal rule, presented a numerical value description of the relationship between sensitivity and specificity for a given diagnostic test. As presented here, a value of 0.5 indicates there is no discrimination within the test and shows any result is essentially the same as a random guess, while a value of 1.0 indicates a perfect test prediction. The use of the 5 marker metabolites achieved the highest AUC value and could significantly increase the diagnostic performance of the metabolic markers (AUC: 0.903, Fig. 4C) with a sensitivity of 96.50% and a specificity of 83%, respectively. Based on the data, it is important to note that the ROC area approaches can perfect accuracy, sensitivity and specificity.

## Discussion

HCC is one of the leading causes of cancer-related death worldwide. Late diagnosis of HCC is the key factor for the poor survival of patients. Current diagnostic methods, the sensitivity of the most common used HCC biomarker, AFP for initial diagnosis and surveillance, is limited to low 70% in clinic^13^. Unfortunately, early stage HCC is usually not noticeable in current clinical practice. A large number of HCC diagnosed are not eligible for surgical intervention, leading to poor prognosis^27^. However, sensitivity of the markers is relatively low, difficult to get outcome immediately and not particularly effective in separating cases of HCC from other disorders. Therefore, biomarkers for the diagnosis of HCC at early stages are essential for the successful management of this disease. Less invasive methods for diagnosis, such as determination of biomarkers from urine, however, would be of significant advantage and useful for primary diagnosis, surveillance and early detection of HCC. Emerging metabolomics provides a powerful platform for discovering novel biomarkers and biochemical pathways to distinguish between diseased and non-diseased status^28^,^29^,^30^.

In this study, metabolic profiling was performed by LC-Q-TOF-MS coupled with multivariate data analysis methods, machine learning approaches, ingenuity pathway analysis and ROC used to select the metabolites to be used for the noninvasive diagnosis of HCC. Results indicate that PCA that is ideally used for analyzing variable-rich datasets, revealed an evident and statistically significant separation between the HCC and control samples. Interestingly, 15 distinct metabolites (VIP > 6) identified in the human urine metabolome, many are in various stages of progress at the HCC. Furthermore, these metabolites were tightly correlated with the bile acid biosynthesis, citric acid cycle, tryptophan metabolism, and urea cycle *etc.* interaction network. Further study of these metabolites may facilitate the development of non-invasive biomarkers and more efficient therapeutic strategies for HCC. The HCC patients were easily distinguished from control subjects by heatmap approach when using autoscaling methods. Such changes are expected to be reflected in wider coverage metabolic profiles, which may in turn be explored as potential biomarkers for HCC assessment and treatment. Overall, these results provided preliminary evidence for these metabolites to be used as biomarkers for the classification of HCC diseases, suggested that metabolomics study is a promising strategy for identifying novel biomarkers of HCC. Pathway and network analyses have both been applied to metabolomic analysis, which vastly extends its clinical relevance and effects. Thus, metabolomics holds promise for early diagnosis, increased choice of therapy and the identification of new metabolic pathways that could potentially be targeted in liver disease. Based on the KEGG, a detailed construction of the the altered bile acid biosynthesis pathways map with higher score is shown in Fig. 3C. These results suggest that these target pathways show the marked perturbations over the entire time-course of HCC and could contribute to the development of HCC. These biochemical changes are helpful to understand the key features of HCC.

The SAM method was used to select the potential biomarkers, palmitic acid, alpha-N-phenylacetyl-L-glutamine, phytosphingosine, indoleacetyl glutamine, and glycocholic acid, were the most significant differential metabolites for the classification of the HCC and controls. To determine whether the metabolic markers that were identified could be used for clinical HCC diagnosis, we performed a preliminary validation using the urine from 15 patients with HCC diseases. Additionally, data show 5 marker metabolites have a better AUC value (0.903) and found that 5 marker metabolites were effective for the discrimination of HCC patients, achieved a sensitivity of 96.5%. Thus, metabolomics has the potential of finding biomarkers for the early diagnosis of HCC and has the capacity to provide a diagnostic tool for the rapid determination of HCC. A metabolomics approach could generate unbiased small molecule metabolic profiles in urine that predict risk for HCC. Through the use of multivariate statistics and machine learning algorithms, the potential of metabolomic analysis has been demonstrated for uncovering biomarkers for specific determination and diagnosis of HCC disease. Our study highlights advantages of metabolomics based HCC diagnostics, and the potential for biomarkers. The size of this study also means it has likely to identify other metabolites that will be important for diagnostics in the future. Once a panel of key biomarkers has been established there is the potential to take metabolomics closer to the bedside and it is the evolution of which will likely be rapid in the next few years.

## Conclusions

Identification of sensitive and specific biomarkers for HCC early diagnosis is of great importance in biological medicine to date. Metabolomics can provide a powerful approach to discover diagnostic and therapeutic biomarkers by analyzing global changes in an individual’s metabolic profile. In this study, we investigated the perturbed metabolic pattern in urine from HCC patients and identified metabolic markers associated with HCC using LC-ESI-QTOF-MS coupled with multivariate statistical analysis. We have identified 15 differential metabolites associated with HCC. More importantly, of 15 differential metabolites, 5 marker metabolites were provided the effective diagnosis for human HCC. Interestingly, the bile acid biosynthesis, citric acid cycle, tryptophan metabolism, and urea cycle metabolism were found that the most altered functional pathway associated with HCC according to ingenuity pathway analysis. A prediction model was developed to indicate HCC, and sensitivity of 96.5%, and specificity of 83% was obtained. Our findings suggest that metabolomic approach is highly effective in aiding biomarker identification. In addition, the more patients included and the detected metabolomic biomarkers make evaluation in further investigations necessary before the significance of our results could be assured.

## Materials and Methods

### Ethical Statement

All healthy volunteers and HCC patients provided informed consent prior to collection of any data. The study was approved by the Ethical Committee of Heilongjiang University of Chinese Medicine and complied with the provisions of the Good Clinical Practice Guidelines and the Declaration of Helsinki.

### Subjects

Early HCC (n = 25) patients and control subjects (n = 12) were recruited in this study, according to the criteria of “International Consensus Group for Hepatocellular Neoplasia” by two independent expert pathologists. The outcomes of Health Survey Questionnaire in patients with HCC and controls were assessed, and the related clinical information including gender, age, body mass index (BMI), basic syndromes of disease and main parameters of liver makers were displayed in supporting information (SI) Table 1. Exclusion criteria are that patients (nonsmoker) had cardiac insufficiency, renal inadequacy, respiratory failure, alimentary tract hemorrhage, or other diseases that will affect the clinical observations and biological indicators. To avoid influence of ageing and gender on metabolomic profiles, subjects in the healthy control cohort were matched each to corresponding subjects in HCC cohorts (age matched within 5 years). Then, a second set of HCC (n = 15) patients and control subjects (n = 10) to be randomly selected and tested using our approach.

### Chemicals and reagents

Acetonitrile, HPLC grade, was obtained from Merck (Darmstadt, Germany); methanol (HPLC grade) was purchased from Fisher Scientific Corporation (Loughborough, UK); water was produced by a Milli-Q Ultra-pure water system (Millipore, Billerica, USA); formic acid was of HPLC grade, and obtained from Honeywell Company (Morristown, New Jersey, USA); leucine enkephalin was purchased from Sigma-Aldrich (St. Louis, MO, USA). All other reagents were HPLC grade.

### Preparation of Urine Samples

Urine was collected in sterile urine containers, pipetted into transport tubes. The subjects were given insulated ice packs in which they were asked to store the urine samples immediately until they were received by the study investigator. On arrival at the laboratory, the samples were centrifuged at 10,000 rpm for 10 min at 4 °C to remove any solid debris. Urine samples were collected after centrifugation at 10,000 rpm for 10 minutes at 4 °C, and the supernatant was transferred to a 1.5 mL polypropylene tube, and then filtered through a 0.22μm microporous membrane), 5 μL of the supernatant were injected into the LC-Q-TOF-MS.

### Metabolic profiling

#### LC-ESI MS analysis

Chromatographic separation was carried out on an ACQUITY BEH C_18_ chromatography column (150 mm × 2.1 mm, 5 μm) with the column temperature of 45 °C, and a mobile phase consisted of phase A (water with 0.1% formic acid) and phase B (acetonitrile containing 0.1% formic acid). The gradient for the urine sample was as follows: 0–5 min, 1–25% B; 5–9 min, 25–50% B; 9–9.1 min, 50–99% B; 9.1–11 min, 99% B; 11–11.1 min, 99–1% B; 11.1–13 min, 1% B. The injection volume was 5 μL with a flow rate of 0.5 mL/min. The eluent was introduced to the mass spectrometer directly. The QC was prepared by pooling together equal volume aliquots of all individual urine samples in order to assess LC-MS system stability.

#### Mass spectrometric conditions

The MS system was operated using the positive-ion (ESI^+^) and negaitive-ion (ESI^–^) mode so as to monitor as many ions as possible, and the mass range was set at 50–1000 m/z in the full scan mode. The optimal capillary voltage was set at 3200 V, and cone voltage at 35 V. Nitrogen was used as the dry gas, the desolvation gas flow rate was set at 500 L/h, and cone gas flow was maintained at 50 L/h. The desolvation temperature was set at 350 °C, and source temperature at 110 °C. The scan time and inter-scan delay were set to 0.4 s and 0.1 s, respectively. Leucine enkaphalin was used as the reference compound ([M + H]^+^ = 556.2771) and [M-H]^−^ = 554.2615) at a concentration of 0.2 ng/mL under a flow rate of 100μl·min^–1 ^for accurate mass calibration in real time. The data were collected in the centroid mode, and the LockSpray frequency set at 10 s and averaged over 10 scans for correction.

#### Multivariate data analysis

Mass chromatograms and mass spectral data were acquired using MassLynx software (Waters Corp.) in centroid format and the multivariate data matrix was analyzed by EZinfo software (Waters Corp., Milford, USA). The unsupervised principal component analysis (PCA) score plots allowed the visualization of data and compared samples between the HCC group and control group. Potential markers of interest were extracted from the combining VIP- plot that was constructed from the loading plots of orthogonal projection to latent structures discriminate analysis (OPLS-DA). With the completion of the OPLS-DA analysis, we can try MetaboAnalyst software version 2.0 to distinguish between control and HCC subjects. Machine learning approaches (heatmaps, random forest) have been used in prediction pattern recognition, exploratory data analysis, and datamining problems. Random Forests algorithm allows for the ability to verify that potential candidates for biomarkers.

#### Identification of Urinary Biomarkers

Exact molecular mass data from redundant *m/z* peaks corresponding to the formation of different parent and product ions were first used to help confirm the metabolite molecular mass. The MassFragment™ application manager (Waters MassLynx v4.1, Waters corp., Milford, USA) was used to facilitate the MS/MS fragment ion analysis process by way of chemically intelligent peak-matching algorithms. The identities of the specific metabolites were confirmed by comparison of their mass spectra and chromatographic retention times with those obtained using commercially available reference standards. Elemental compositions were derived considering a mass error less than 5 ppm.

#### Metabolic Pathway Analysis

The construction, interaction and pathway analysis of potential biomarkers of HCC patients was performed with Metabolite Set Enrichment Analysis (MESA) based on the updated database source to identify the top altered pathways analysis & visualization. The possible biological roles was also evaluated by the enrichment analysis using the MetaboAnalyst software 2.0. MassTRIX (http://metabolomics.helmholtz-muenchen.de/masstrix/), a web-based tool designed to assign ions of interest from a metabolomics experiment to annotated pathways without any systematic identification, was used to identify the affected metabolic pathways.

#### Data analysis

SPSS software 17.0 for Windows was used for the statistical analysis. The data were analysed using the Wilcoxon Mann-Whitney Test, with p < 0.05 set as the level of statistical significance. MetaboAnalyst software was used for the HCA and significance analysis for microarrays (SAM). Diagnostic model was constructed with the marker metabolites, using linear discrimination analysis method. The classification performance (specificity and sensitivity with the highest accuracy) was assessed by AUC value of the ROC curves.

## Additional Information

**How to cite this article**: Liang, Q. *et al.* Phenotypic Characterization Analysis of Human Hepatocarcinoma by Urine Metabolomics Approach. *Sci. Rep.* **6**, 19763; doi: 10.1038/srep19763 (2016).

## Supplementary Material

Supplementary Information
