# Supplementary material for: Phenotypic Characterization Analysis of Human Hepatocarcinoma by Urine Metabolomics Approach
Source: Sci Rep. 2016 Jan 25;6:19763. doi: 10.1038/srep19763 (PMC4726192; doi:10.1038/srep19763)
Supplement: Supplementary Information [file srep19763-s1.pdf]

Phenotypic Characterization Analysis of Human Hepatocarcinoma by Urine Metabolomics Approach

Qun Liang[1]*, Han Liu[2], Cong Wang[1], Binbing Li[1]

1. First Affiliated Hospitaly, Heilongjiang University of Chinese Medicine, Heping Road 24, Xiangfang District, Harbin 150040, China

2. Simon Fraser University (SFU), Burnaby, British Columbia, Canada.

Correspondence

Prof. Qun Liang

First Affiliated Hospital

Heilongjiang University of Chinese Medicine

Heping Road 24,

Xiangfang District,

Harbin 150040, China

Tel. & Fax +86-451-86053141

Email: qunliang1970@126.com

Table 1. Clinical characteristics of the subjects at baseline.

Samples	HCC	Control
Sample No.	25	12
Age	38±4	40±3
Sex (F/M)	12/13	6/6
BMI(kg/m2)	21.77±2.56	22.25±1.91
ALT (U/L)	110.46±49.35	56.78±27.13
AST (U/L)	90.10±52.45	37.07±26.92
GGT(U/L)	76.18±17.72	34.27±9.34
Median tumor size (cm)	<2 cm	-
AFP(ng/mL)	109.22±3.76	1.64±0.89

Table 2. Primary metabolites differentially expressed in urine of HCC and normal control patients.

No.	VIP	Rt	$[M+H]^+$	$[M-H]^-$	Formula	Metabolites	HMDB	PubChem
1	10.3182	4.64	257.0823		$C_{16}H_{34}O_2$	Palmitic acid	HMDB00220	985
2	9.55195	3.84	265.1887		$C_{13}H_{16}N_2O_4$	Alpha-N-Phenylacetyl-L-glutamine	HMDB06344	92258
3	7.5964	5.22	318.2927		$C_{18}H_{40}NO_3$	Phytosphingosine	HMDB04610	122121
4	7.18613	3.81	304.1372		$C_{15}H_{19}N_3O_4$	Indoleacetyl glutamine	NA	NA
5	6.88439	8.46	466.3082		$C_{26}H_{44}NO_6$	Glycocholic acid	HMDB00138	439604
6	6.68089	3.83	179.0514		$C_9H_9NO_3$	Hippurate	HMDB00714	464
7	6.51659	4.29	220.9994		$C_{11}H_{14}N_2O_3$	5-Hydroxy-L-tryptophan	HMDB00472	144
8	6.33054	2.61	144.0076		$C_5H_5O_5$	2-Oxopentanedioate	HMDB00208	51
9	6.25576	4.53	516.2921		$C_{26}H_{46}NO_7S$	Taurocholic acid	HMDB00036	440567
10	6.07649	4.43	393.2948		$C_{24}H_{41}O_4$	Chenodeoxycholic acid	HMDB00518	10133
11	8.5538	6.5		539.2488	$C_{27}H_{40}O_{11}$	Cortolone-3-glucuronide	HMDB10320	125853
12	7.1982	7.19		285.0062	$C_{19}H_{24}O_2$	Androstenedione	HMDB00053	6128
13	6.4612	5.24		252.1392	$C_{13}H_{17}NO_4$	3-Indolecarboxylic acid	HMDB03320	69867
14	6.1047	6.66		541.2601	$C_{27}H_{42}O_{11}$	Cortolone-3-glucuronide	HMDB10320	125853
15	6.0566	2.44		195.0496	$C_{10}H_{12}O_4$	Homoveratric acid	HMDB00434	7139

Table 3: Pathway-associated metabolite sets for HCC from Metabolite Set Enrichment Analysis.

No.		Total	Expected	Hits	Raw p	p-value
1	Bile acid biosynthesis	49	0.772	3	0.037	0.00
2	Citric acid cycle	23	0.362	1	0.309	0.01
3	Tryptophan metabolism	34	0.536	1	0.424	0.02
4	Urea cycle	20	0.315	1	0.275	0.04
5	Phenylacetate metabolism	4	0.063	1	0.062	0.06
6	Alanine metabolism	6	0.095	1	0.091	0.06
7	Malate-aspartate shuttle	8	0.126	1	0.120	0.09
8	Glucose-alanine cycle	12	0.189	1	0.175	0.12
9	Glycerolipid metabolism	13	0.205	1	0.188	0.12
10	Androgen and estrogen metabolism	17	0.268	1	0.239	0.17
11	Ammonia recycling	18	0.284	1	0.251	0.19
12	Glutamate metabolism	18	0.284	1	0.251	0.22
13	Insulin signalling	19	0.299	1	0.263	0.28
14	Fatty acid elongation in mitochondria	26	0.410	1	0.343	0.38
15	Gluconeogenesis	27	0.425	1	0.353	0.47
16	Fatty acid metabolism	29	0.457	1	0.374	0.47

Table 4: Location-based metabolite sets for HCC from Metabolite Set Enrichment Analysis.

No.		Total	Expected	Hits	Raw p	p-value
1	Liver	234	4.06	4	0.618	0.01
2	Hepatocyte	1	0.02	1	0.017	0.03
3	Fibroblasts	183	3.17	4	0.395	0.03
4	Kidney	164	2.84	2	0.815	0.05
5	Platelet	108	1.87	2	0.580	0.12
6	Epidermis	100	1.73	2	0.535	0.13
7	Thyroid gland	12	0.21	1	0.191	0.14
8	Myelin	26	0.45	1	0.370	0.22
9	Skeletal muscle	45	0.78	1	0.556	0.33
10	Membrane	50	0.87	1	0.595	0.37
11	Endoplasmic reticulum	53	0.92	1	0.617	0.42
12	Bladder	92	1.59	1	0.820	0.48
13	Muscle	101	1.75	1	0.850	0.60
14	Brain	122	2.11	1	0.903	0.62
15	Intestine	145	2.51	1	0.940	0.78

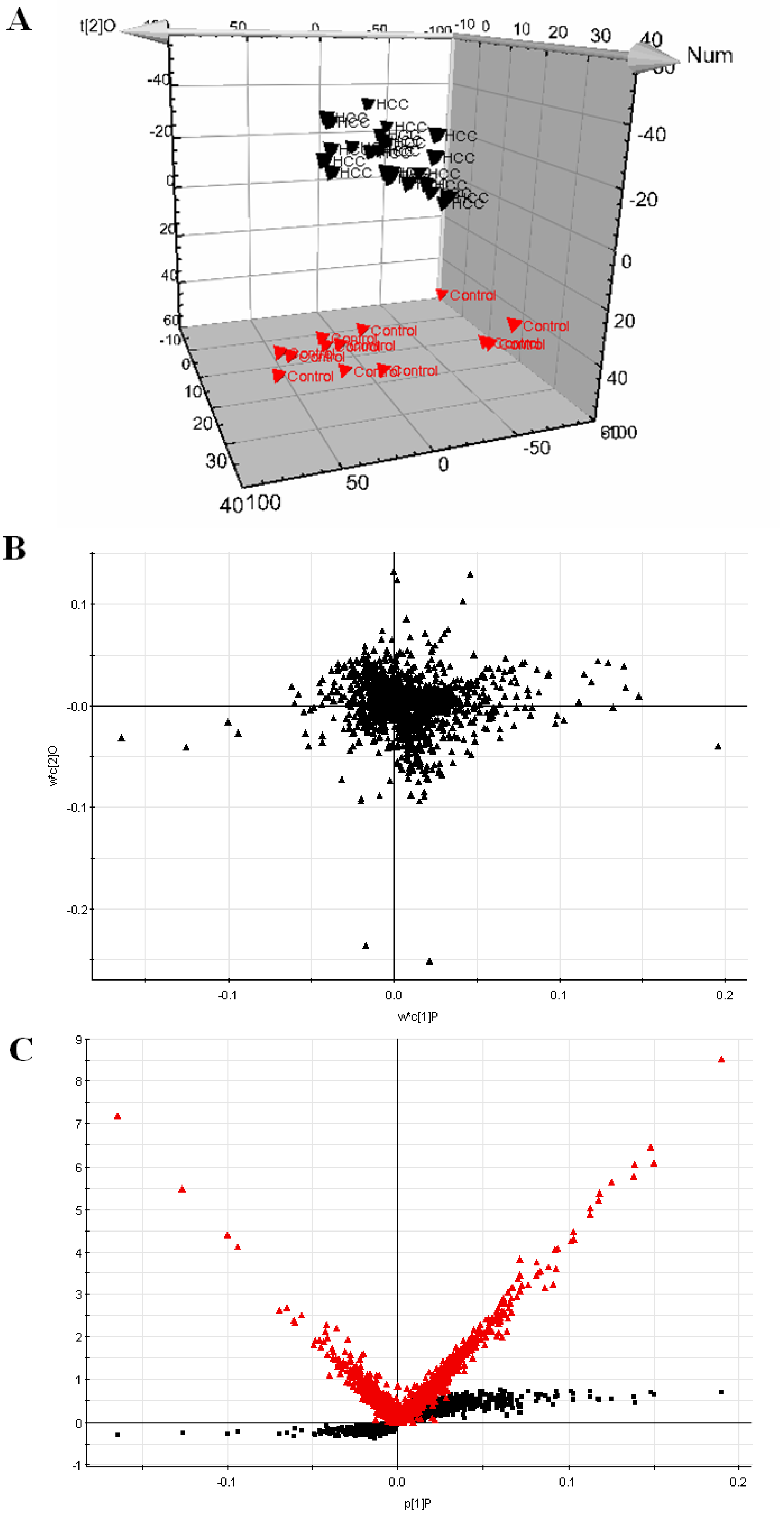

Fig. S1. Metabolomic profiling of HCC in positive mode. 3-D of PCA model for HCC group (A). Loading plot of OPLS-DA of HCC in positive mode (B). Panel C shows the combination of S- and VIP-score plots constructed from the supervised OPLS analysis of urine (ESI^- mode) mode (C).
